# The Incidence of Complications of Dental Trauma and Associated Factors: A Retrospective Study

**DOI:** 10.1155/2020/2968174

**Published:** 2020-03-11

**Authors:** Ines Kallel, Nabiha Douki, Syrine Amaidi, Faten Ben Amor

**Affiliations:** ^1^Department of Dental Medicine, Sahloul Hospital, Sousse, Tunisia; ^2^University of Monastir, Faculty of Dentistry, Research Laboratory Oral Healh and Rehabilitation (LR12ES11), 5000 Monastir, Tunisia; ^3^Department of External Dental Consultation, Clinic of Monastir, Monastir, Tunisia

## Abstract

**Objective:**

The aim of this work was to study the incidence of complications of dental traumatisms and look for associations between factors related to trauma and the occurrence of complications. *Materials and Methods*. It is a longitudinal retrospective study on a sample of 125 traumatized teeth. The sample is taken from patients consulting the dentistry service at the hospital Sahloul Sousse between 2014 and 2017. Criteria for including a patient were presence of a permanent incisor affected by a subluxation, intrusion, lateral luxation, extrusion, or avulsion injuries associated or not with concomitant dentoalveolar injuries. Data were collected using a questionnaire. The information about etiology of trauma, delay of consultation, orientation of the patient, kind of injury, and emergency treatment and complications were obtained from the patients' records.

**Results:**

The incidence of complications was 8%: external root resorption was present in 70% of cases, surface resorption was observed in 10% of cases, and replacement resorption in 10%, ankylosis in 10%. About pulpal complications, pulp necrosis was found after 4 weeks of follow-up, as well as the internal root resorption after one year. The most common cause of the trauma was the fall (40%). The majority of patients came for emergency consultation within “1 to 3 days,” and the coronary fracture without pulp exposure was the first diagnosis (20.60%). Statistical analysis showed no significant relationship between the cause of the trauma and the complication (*P*=0.577) and between the delay of consultation and complication (*P*=0.577) and between the delay of consultation and complication (*P*=0.577) and between the delay of consultation and complication (

**Conclusion:**

Treatment of dental injuries is usually delayed and not given as much attention as general medical treatment that can explain the occurrence of pulpal and periodontal complications. Immediate consultation and treatment could improve long-term prognosis of the injured tooth.

## 1. Introduction

Traumatic dental injuries (TDIs) of permanent teeth occur frequently in children and young adults. Crown fractures and luxations are the most commonly occurring ones of all dental injuries [1]. Proper diagnosis, treatment planning, and follow-up are important for improving a favorable outcome. The objective of this study was to evaluate the incidence of complications of dental trauma on a sample of 125 traumatized teeth and the effect of some variables of injury like etiology of trauma, diagnosis, delay of consultation, and orientation of patients and treatment variables like treatment of emergency and complications.

## 2. Materials and Methods

It is a longitudinal retrospective study. A sample of 125 traumatized teeth was drawn from the patients who attended the department of dentistry at the hospital of Sahloul Sousse (Tunisia) between 2014 and 2017. Patients were referred from dentists or medical practitioners in the Department of Emergency or Department of Oral and Maxillofacial Surgery or by themselves. Data were collected using a questionnaire. The data collected at the emergency visit included etiology of trauma, delay of consultation, orientation of the patient, and type of injury. The emergency treatment was reported, and the information collected at follow-up visit includes the following: pulp necrosis, internal root resorption, canal obliteration, external root resorption, surface resorption, replacement resorption, and ankyloses. The classification of TDIs proposed by Andreasen and Andreasen based on a system adopted by the World Health Organization [2] was used to classify the injuries. The diagnosis was based on both clinical and radiological findings. Data analysis was undertaken using SPSS for Windows 20.0 statistical software (SPSS, Inc., Chicago, IL, USA). Chi-squared tests were used to compare qualitative data and determine statistical significance at level of 5%. The distribution of the parameters on which our tests were carried out follows the normal distribution.

## 3. Results

### 3.1. Etiology of Trauma

**Figure 1:**
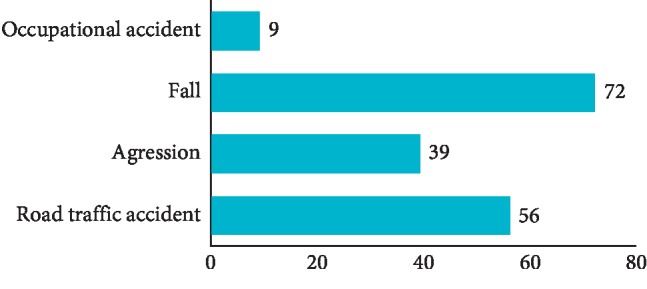
Etiology of trauma.

The most common cause of the trauma was due to fall in 40% of cases, followed by road traffic crash (33.12%). Violence was responsible in 21.25% and occupational accidents in 5.63% (Figure 1).

The association between the cause of the trauma and the complication occurrence was found at *P*=0.577(>0.05), so there was no relationship between the two variables.

There was a significant relationship (*P*=0 < 0.05 between age and cause of trauma.

### 3.2. Delay between the Date of the Consultation and the Date of the Trauma

**Figure 2:**
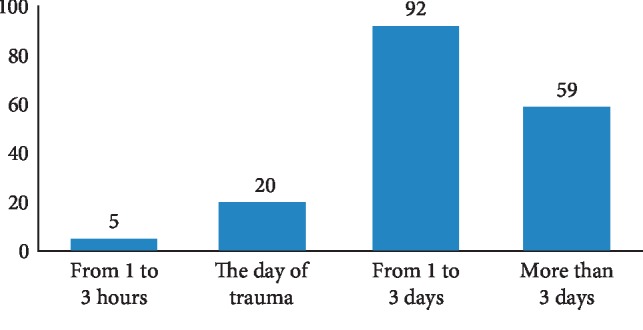
Delay between consultation and trauma.

Time between the date of the consultation and the date of the trauma differs between patients. The majority of patients came for emergency consultation within “1 to 3 days.” However, the delay “from 1 to 3 hours” remains the least represented (3.12%) (Figure 2).

### 3.3. Patient Orientation

**Figure 3:**
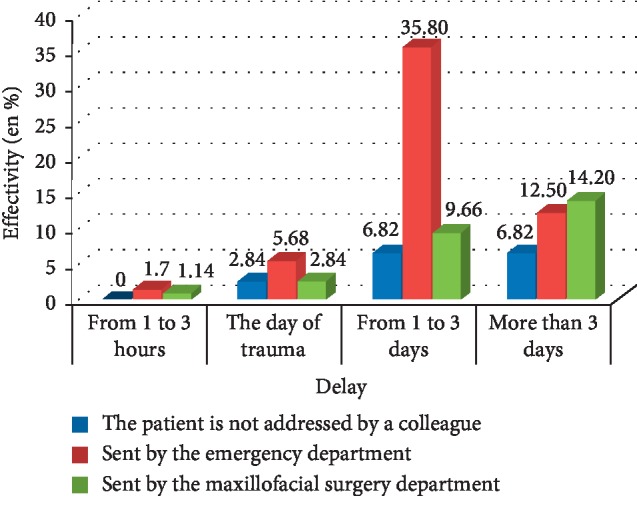
Distribution of patients according to their consultation delay and orientation.

More than half of the patients were referred by the Emergency Department. 26.25% of cases were referred by the Maxillofacial Surgery Department, and 18.75% of cases came to consult by themselves. The majority of patients referred by the Emergency Department were referred within 1–3 days, while those collected by the Maxillofacial Surgery Department were referred after more than 3 days of the date of trauma. The frequency of patients who consult by themselves remains minor (Figure 3).

The chi ^2^ test allowed us to look that there was a statistically significant association between consultation time and patient orientation (*P*=0.009).

### 3.4. Diagnosis

**Figure 4:**
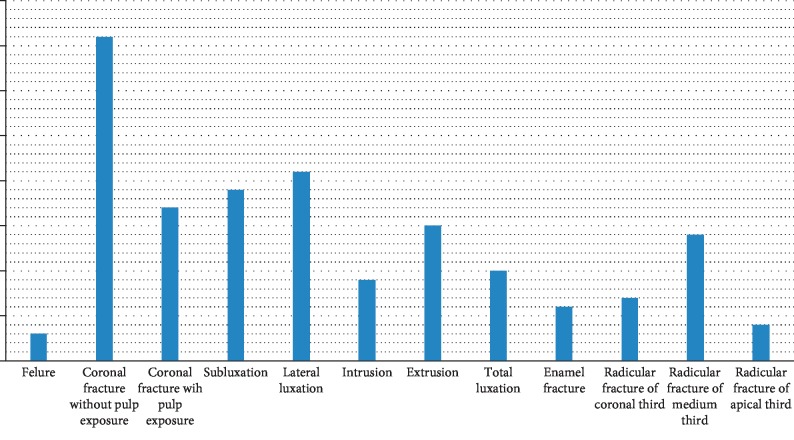
Distribution of patient according to diagnosis.

The coronary fracture without pulp exposure was the first diagnosis with 20.60% of cases, followed by lateral luxation with 12.50% and subluxation with 10%. The rest of the diagnosis are well detailed in Figure 4.

### 3.5. Emergency Treatment

The emergency treatment was related to the diagnosis. For the enamel fracture, the treatment was divided between laying of varnish (60%), restoration with composite resin (20%), and ameloplasty (20%). For coronary fracture without pulp exposure, the use of a liner based on calcium hydroxyde and glass ionomer cement was the most used therapeutic solution (57.6%). On the other hand, bonding of the fragment was rarely used (6%). For coronary fractures with pulpal exposure, the emergency treatment was endodontic in 53% of cases, direct capping in 26% of cases, and pulpotomy in 21% of cases. In case of a root fracture of the 1/3 apical, abstention was the therapeutic of choice in 80% of the cases: for root fracture of 1/3 median, it was opted for a reduction followed by splinting in 47% of the cases and the extraction in 33.4% and abstention for the rest of the cases. For cervical fracture with a communication with oral cavity, the treatment was to remove the coronal fragment and to put a cavity liner + glass ionomer cement (42%) or extraction (28.60%). If cervical fracture was without communication with oral cavity, a splint was placed for 8 weeks. As for extrusion, the repositioning of the tooth + splint for 2 weeks was the most frequent emergency act with 85.70%, while extraction was chosen in 14.30% of cases. The surgical repositioning + flexible splint for 4 weeks was the emergency act for intrusion in 62.50% of the sample. The rest of the patients did not receive any treatment on the day of the emergency consultation hoping for a spontaneous eruption. Digital repositioning + flexible splint 3 to 4 weeks was the main emergency treatment in case of lateral luxation (95%). The predominant emergency treatment following total dislocation consisted in immersing the tooth in a solution of 2% sodium fluoride for 20 minutes + endodontic treatment in extra oral + reimplantation of the tooth + splint for 4 weeks or immersion of the tooth in 2% sodium fluoride solution for 20 minutes + reimplantation + splint for 4 weeks and endodontic treatment reported for the second appointment one week later (if extra oral time >60 min). If extra oral time was <60 min, reimplantation + splint for 2 weeks + endodontic monitoring was chosen.

### 3.6. First Monitoring Session

**Figure 5:**
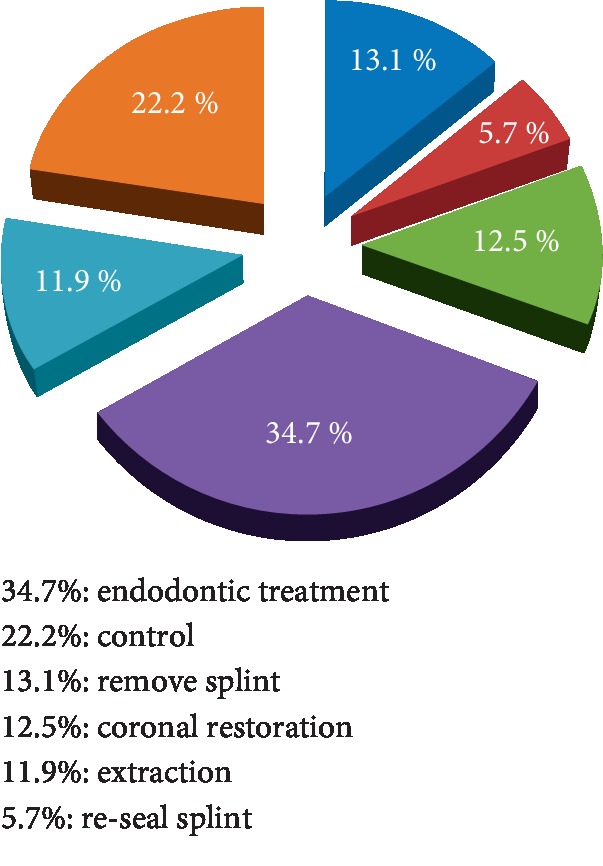
Operations at the first monitoring session.

At the first monitoring session, the operations differed according to the type of traumas, the pulp vitality, and the integrity of the periodontal system. Endodontic treatment was at the top of the list of dental care with 34.7%, followed by a simple control session with 22.2% during which the appropriate control tests were carried out, splint removal on 13.1%, coronal restoration on 12.5%, and extraction on 11.9%. Unsealed splint was reconstructed for 5.7% (Figure 5).

### 3.7. Complications

**Figure 6:**
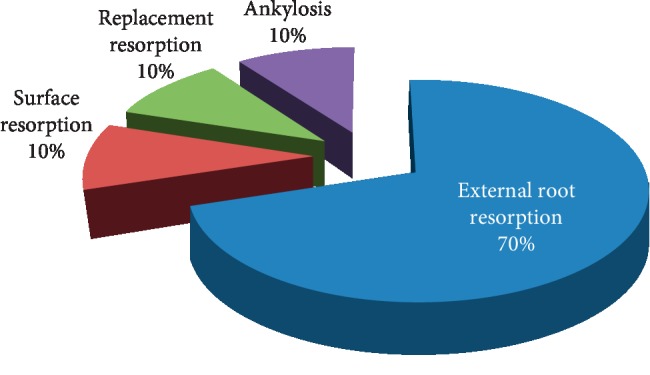
Periodontal complications.

Referring to our sample, 8% of the cases had subsequent complications: periodontal complications (83%) and pulp complications (17%). About the periodontal complications, the external root resorption was present in 70% of cases, almost half of which was recorded after 4 weeks; surface resorption was observed in 10%, replacement resorption in 10%, and ankylosis in 10% (Figure 6).

**Figure 7:**
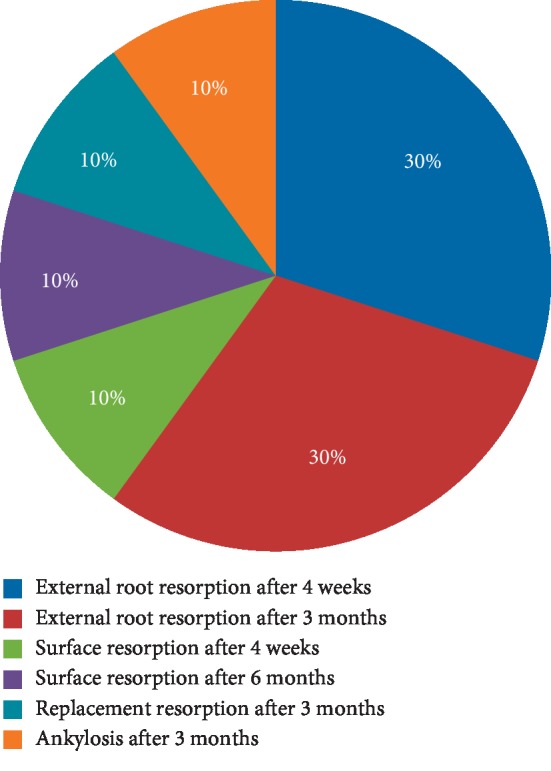
Onset of periodontal complications.

The onset of periodontal complications with time is reported in Figure 7.

About pulp complications, pulp necrosis was observed after 4 weeks of follow-up and internal root resorption after one year.

### 3.8. Relation between Complications and Delay of Consultation

**Figure 8:**
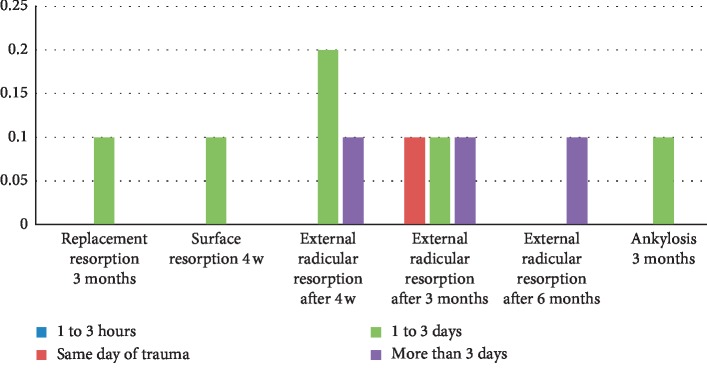
Relation between complications and delay of consultation.

In our study, patients who had emergency consultations within 1 to 3 hours after trauma had no complications. However, 10% of patients consulting on the same day of the trauma presented an external root resorption at 3 months. The longer the delay of consultation, more complications occurred. Thus, for a period of 1 to 3 days, the patients underwent ankylosis at 3 months, replacement resorption at 3 months, surface resorption at 4 weeks, and external root resorption at 4 weeks or at 3 months. For patients who came for consultation 3 days after trauma, they subsequently suffered from external root resorption at 4 weeks, 3 months, or 6 months (Figure 8).

The association between the delay of consultation and complication was found at *P*=0.143 (>0.05), which means the absence of relationship between the two variables.

### 3.9. Relation between Complications and Diagnosis

**Figure 9:**
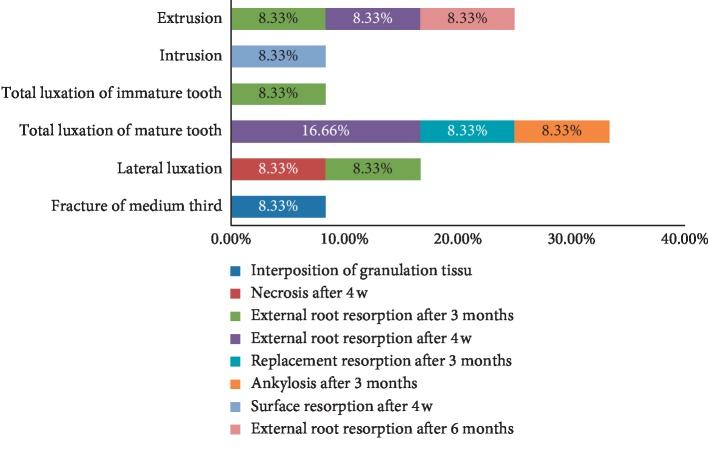
Relation between complications and diagnosis.

The complications observed were related to specific types of trauma. Indeed, total dislocation generated the highest percentage of subsequent complications. In addition, in cases of total dislocation of mature teeth, the complications were distributed as follows: 16.66% of external root resorption after 4 weeks, 8.33% of resorption of replacement after 3 months and 8.33% of ankylosis after 3 months. In case of a total dislocation of immature teeth, 8.33% of external root resorptions was seen after 3 months. Regarding extrusion, it generated a single type of complication, the external root resorption which was seen after 4 weeks, after 3 months, and after 6 months with an equal occurrence frequency. Regarding lateral dislocation, the complications observed were necrosis after 4 weeks and external root resorption after 3 months with 8.33% for each. Finally, there is the intrusion. The intrusion generated surface resorption after 4 weeks with 8.33%; root fracture at the middle 1/3 caused internal root resorption after one year with 8.33%. The following figure illustrates these statements (Figure 9).

The association between the diagnosis and the complication occurrence was found at *P*=0.47 (>0.05), so there was no relationship between the two variables.

## 4. Discussion

### 4.1. Etiologies of Trauma

The etiology of dental traumatisms was different: in our sample, the most frequent cause is the fall, with 40% of the trauma, then the road accident with 33.12%, then the aggression with 21.25%, and finally the working accident at 5.63%. These results are similar with those collected in the literature. In the study of Bakland and Andreasen [1], the effect and impact of sports accidents in dentoalveolar trauma (almost in 49% of cases) are highlighted. Demars-Fremault and Michela [3] concluded that approximately 28% of accidents occur at school, 27% of domestic accidents, 21% of sports accidents, 11% of acts of violence, and 11% of road accidents. In our study, there was a significant relationship between age and cause of trauma (*P*=0 < 0.05). Indeed, the fall is the main cause of trauma in children in the home environment and the school environment, and in adolescents during sports activities. This is confirmed by several studies such as the study reporting the prevalence of traumatic dental injuries in children and adolescents in Romania, between 2003 and 2011 [4].

### 4.2. Delay between the Date of the Consultation and the Date of the Trauma

After trauma, a dental consultation of patients does not represent the first priority. Indeed, almost half of the cases treated in the dental services of the Sahloul hospital consult after a variable period of 1 to 3 days compared with 35% of them who are consulted after 3 days. Nevertheless, the time from 1 hour to 3 hours does not exceed 3.12% of the sample. This finding is in agreement with the study of Oulis and Berdouses [5], who observed that 68% of their patients in Athens presented for treatment on the 3rd day. Also Gábris et al. [6] in Budapest showed that 77% of the cases presented for treatment in the first 3 days.

### 4.3. Patient Orientation

Several parameters and factors guide patients to consult, such as the age of the patient, the severity of the accident, and the number of injured teeth. Based on the results of our study, we find that almost half of the patients was referred by the emergency department, while the cases sent by the maxillofacial surgery department were only 26.25%. The rest consult on their own initiative. These results appear quite real since patients are, of course, moving towards the emergency services as a first resort in case of accident or injury. Patients with mild-to-moderate trauma, such as contusion or coronary cracks, do not necessarily call for emergency services and consult directly their treating dentists. However, when the general lesions are aggravated, such as certain orofacial head injuries, the patient is systematically referred to a specialized department (neurosurgery and maxillofacial surgery) and proceeds to a more advanced level of treatment. It will then be sent to the dentistry department in the second time, so this delay could have adverse consequences on the prognosis especially for the cases of total dislocations. A statistically significant relationship was found between consultation time and patient referral (*P*=0.009 < 0.05). This association could have several explanations. First of all, these patients are usually multitraumatized, so initially consult the emergency department where the vitality is the priority. Then, depending on the severity of the patient's conditions, the dental care is postponed. In addition, victims of accidents are treated with analgesics and anti-inflammatories that will partially or completely mask the dental pain. Thus, the patient will not feel the need to consult urgently and they will come to the service only when there is a complication. The time interval between the moment of the trauma and the date of consultation is a decisive element in our therapeutic choice and it influences the prognosis.

### 4.4. Diagnosis

In our study sample, the coronary fracture without pulp exposure was the most represented diagnosis with 20.60%, followed by lateral dislocation with 12.50% and then subluxation with 10%. The least represented diagnosis was the total dislocation of the mature teeth. This result is similar to other studies like the study done in the Department of Dentistry at the Pilsen Faculty Hospital [7] where dentin-enamel fractures (26.2%) and lateral dislocations (23.3%) were the most frequent. Another study in Brazil in 2017 [8] found uncomplicated coronary fracture as the most common with 52.6% and then subluxation with 25%. In addition, the expulsion of permanent teeth is infrequent and accounts from 0.5 to 16%. The type of trauma varies mainly with age. Thus, for patients aged less than 10 years, the most marked types of trauma were coronary fracture without pulp exposure with 30% and subluxation with 16.6%. These results are similar to those found in a study of a total of 4956 children aged 6–12 in the Department of Pediatric Dentistry Center for Dental Sciences in Ankara, showing that the most common type of lesion was enamel fracture (44.6%) [9].

In mixed dentition, the alveolar bone becomes more compact and more resistant to lateral and axial movements. Fractures of the dental organs became more frequent with root elongation and bone densification. From adolescence, the definitive dentition is built and the consequences of a trauma are heavier. For the second and third decades, the most common diagnosis in this population was coronary fracture without pulpal exposure, lateral dislocation, and contusion. This is confirmed by several studies. With age, the frequency of coronary and root fractures increases. In fact, the adult shares many risks (road accident and violence), to which must be added the physiological aging of the teeth and the reduced supportive tissues [10]. Coronary fractures as well as enamel fractures are the most common type of trauma observed in permanent dentition, unlike the temporary dentition, which has been shown to be predominantly dislocated due to the plasticity of bone structures [11].

### 4.5. Emergency Treatment

In our study, 50% of the patients coming to consult for cracks were treated with a varnish application, whereas the practitioners preferred to abstain for the rest of the observed cases. As for enamel fractures, 60% of patients were treated with a varnish while the other 40% was divided between ameloplasty and composite restoration. Coronal fracture without pulpal exposure was the most common type of trauma in our sample. More than half of the patients received a pulp capping with calcium hydroxide and coronal obturation with glass cement ionomer, compared with 36.4% who were treated by composite restoration; the collage of the fragment was practiced only for 6% of patients. A coronary fracture with pulpal exposure requires careful and appropriate management. The decision of the treatment depends essentially on the stage of root evolution. In our sample, the endodontic treatment was the main act of emergency for the mature teeth with 53.31%, while only 13.33% of cases with pulp exposure received direct pulp capping. These results are in agreement with the study by Andreasen et al. [2]. On the other hand, for immature teeth, emergency treatment differed between direct pulp capping (13.33%), partial pulpotomy (13.33%), and total pulpotomy (6.7%). Radicular fractures most often result from horizontal shock at different levels: apical 1/3, middle 1/3, and 1/3 coronary. The treatment depends on two factors: the degree of maturation of the apex and the level of the fracture line. The root fracture of 1/3 apical is the most favorable situation, because in the majority of cases, neither mobility nor displacement of the fragments is observed. Only a regular monitoring is programmed. This is in agreement with the results of our study. Practitioners chose abstention for 80% of patients consulting for a root fracture of 1/3 apical, and the remaining 20% were treated by splinting. The root fracture of the middle 1/3 is the most common. Treatment consists of reducing the fracture line and splint. This was recorded in 46.6% of cases. Thus, the shorter the time between trauma and consultation, the easier the reduction of the fracture. In 33.4% of patients, the affected tooth was extracted. The rest did not receive any treatment during the emergency consultation. This can be explained by the fact that some patients have suffered serious general injuries, such as head and facial trauma. For cervical fracture, two situations arise: with or without communication of the fracture line with the oral cavity. If there is a communication, we can either remove the coronal fragment and to put a cavity liner + glass ionomer cement (42% of cases) or extraction (28.60%); otherwise (no communication with the oral cavity), a splint was placed during 8 weeks. For contusion, no treatment was performed during the emergency consultation. Only one prescription of a soft diet for 2 weeks was required. When the impact of the shock is more serious than the previous cases, some periodontal fibres may be broken and subluxation occurs. In this case, the treatment consisted in the prescription a soft diet and splint for 2 weeks for half of patients while for the remaining half only a soft diet was prescribed. Extrusion usually results from an oblique trauma that moves the tooth out of its socket. The periodontal ligament and the neurovascular system of the pulp are severely affected. The repositioning of the tooth in its socket and splint for 2 to 3 weeks was the most important emergency act in our sample with 85.70%; extraction remains the last resort, but it was practiced in 14.3% of cases. Intrusion is the most severe form of dental trauma. The most often axial shock push the tooth into its socket, usually resulting in perforation. The treatment depends essentially on the stage of root development. Surgical repositioning and splint for 4 weeks was the predominant act of urgency with 62.50% of the sample, while abstain hoping for a spontaneous eruption in the remaining 37.5%. In case of lateral luxation, emergency treatment consisted of repositioning the tooth in its original position, associated with splint for 4 weeks at least. In our sample, the digital repositioning and splinting for 3 to 4 weeks was the main emergency treatment in 95% of cases. Total dislocation is the complete movement of the tooth out of the socket. The repair of such trauma depends on the pulpal survival and healing of the periodontal ligament. The determining factor is the extraalveolar time. Reimplantation in the accident site is recommended. In case of impossibility, it is recommended to submerge the tooth in a suitable medium (milk and water). In our study, total dislocation was the least common type of trauma. The immersion of the tooth in 2% sodium fluoride solution for 20 minutes, endodontic treatment in extra oral, tooth reimplantation, and splint for 4 weeks were the emergency acts observed in 60% of cases. This treatment is generally indicated when the extraalveolar time is greater than 60 minutes. The emergency treatment of the rest of the patients was the immersion of the tooth in 2% sodium fluoride solution for 20 minutes, re-implantation, splint and the endodontic treatment later or re-implantation and splint without immediate endodontic treatment when the extra alveolar time is less than 60 minutes. Indeed, extra alveolar time is a major factor in the prognosis of the reimplantation of avulsed teeth. In fact, data from several studies show that reimplantation must be immediate (within 5 minutes) for regeneration of the periodontal ligament and restoration of normal function [12]. In case of late reimplantation, even if the tooth is immersed immediately in a suitable fluid, the periodontal ligament become weakened and lose their ability to transform into fibroblasts and perform the normal functions of periodontal cells. The healing is done by repair, and regeneration of the LPD is weak or absent. When the tooth is immersed in a suitable fluid within 15 minutes of avulsion, some of the LPD and cement cells will survive and contribute to regeneration [7, 13].

### 4.6. Act of the First Monitoring Session

In our sample, 85% of patients returned for further treatment at the first follow-up session. This number seems to decrease from one control appointment to reach 9.6% at the fourth session and 1.2% at the fifth session. This can be explained by the fact that the majority of patients relieved of their pain by the emergency medication and find it unnecessary to continue care. This complicated the work of the practitioners, who must explain to the patient the importance of the therapeutic gesture in the relief of their pain and also the treatment of its cause and the complications that may occur in case of no treatment. It is also important to explain to parents the importance of the follow-up. The absence of care appointments can lead quickly to the appearance of sequels difficult to manage by the practitioner. These sequels will have significant consequences for the durability of these traumatized teeth [14].

### 4.7. Complications

Referring to our sample, 8% of the cases studied had subsequent complications, 83% of which were periodontal complications. Pulpal complications are the least represented and do not exceed 17%. These results differ from those obtained in other similar studies conducted by the Department of Dentistry of the Pilsen Faculty Hospital [15], where pulpal complications predominated. Pulp necrosis was observed in 239 teeth (26.9%). It was the most common posttraumatic complication in all types of dental trauma. Indeed, teeth with complete root had a higher prevalence of pulpal necrosis than teeth with incomplete root formation in all types of dislocation. In this sense, it can be deduced that the majority of cases observed with necrosis in specific age groups were adolescents and adults. Since periodontal complications are the most reported in our study, external root resorption was the main complication with 70%. This type of complication is particularly present with total dislocation, lateral dislocation, and extrusion. Several studies reveal results close to those we found. Thus, the external root resorption concerned by the trauma can be of different natures: surface, replacement, or inflammatory. Therefore, early detection and rapid initiation of appropriate therapy is essential for a good prognosis of the injured tooth. A study on the resorption according to the type of trauma gives the following results: out of 45 cases of resorption, 9 cases were associated with dislocations (20%) and 36 with avulsions (80%). 30 cases of inflammatory resorptions have been described, and 15 cases of replacement resorptions have been observed [16, 17]. Other publication found that resorption after contusion or subluxation occurred in only 3% of cases [18]. On the other hand, in the case of extrusion, resorption occurred in 15% of cases. This percentage increases again up to 30% in case of lateral dislocations [19]. Finally, more recently, a study confirmed that external resorption is more frequently associated with intrusive dislocations (93%), followed by avulsions (89%), lateral dislocation (80%), and extrusive dislocation (77%) [20]. Among the types of trauma, replacement resorptions were observed more often in cases of dental expulsion followed by reimplantations (87%). The explanation of this difference in resorption percentage lies in the fact that, during an extrusion, the pulp is strongly injured by stretching and then immediate rupture of the apical vasculonervous bundle, and the pulp necrosis occurs quickly. In the case of luxation, the pulp necrosis occurs less rapidly and is prone to bacterial infections, and chronic inflammation may occur. The lesion of the outer precement, or the inner, preplant protective layer can then allow the clastic cells to resorb the root at the point of impact of this root in its alveolus [20].

## 5. Conclusion

In our study, dental trauma caused both periodontal and pulp complications. Being the longer the delay of consultation the more complications accumulate; the dental care cannot be postponed and needs immediate intervention. The time interval between the moment of the trauma and the date of consultation is a decisive element in the therapeutic choice and influences the prognosis.

## Data Availability

Previously reported data used to support this study are included within the article. The prior studies are cited at relevant places within the text as references.
